# Experimental Study on the Electrochemical Anti-Corrosion Properties of Steel Structures Applying the Arc Thermal Metal Spraying Method

**DOI:** 10.3390/ma7127722

**Published:** 2014-12-03

**Authors:** Hong-Bok Choe, Han-Seung Lee, Jun-Ho Shin

**Affiliations:** 1 Department of Architectural Engineering, Hanyang University, 1271 Sa 3-dong, Sangrok-gu, Ansan 426-791, Korea; E-Mail: hongbokchoe@gmail.com; 2 Department of Architectural Engineering, Gachon University, 1342 Seongnamdaero, Sujeong-gu, Seongnam-si, Gyeonggi-do 461-701, Korea; E-Mail: jhshin0720@gachon.ac.kr

**Keywords:** arc thermal metal spray, electrochemical technique, anti-corrosion, steel structures

## Abstract

The arc thermal metal spraying method (ATMSM) provides proven long-term protective coating systems using zinc, aluminum and their alloys for steel work in a marine environment. This paper focuses on studying experimentally the anti-corrosion criteria of ATMSM on steel specimens. The effects of the types of spraying metal and the presence or absence of sealing treatment from the thermal spraying of film on the anti-corrosion performance of TMSM were quantitatively evaluated by electrochemical techniques. The results showed that ATMSM represented a sufficient corrosion resistance with the driving force based on the potential difference of more than approximately 0.60 V between the thermal spraying layer and the base substrate steel. Furthermore, it was found that the sealing treatment of specimens had suppressed the dissolution of metals, increased the corrosion potential, decreased the corrosion current density and increased the polarization resistance. Metal alloy Al–Mg (95%:5%) by mass with epoxy sealing coating led to the most successful anti-corrosion performance in these electrochemical experiments.

## 1. Introduction

A hot dip galvanizing (HDG) method using zinc (Zn) and a heavy-duty coating method that applies the spraying of zinc-rich paint on primer and that combines epoxy resin and fluoride resin have been largely favored [1,2,3]. However, these methods showed several problems. In particular, the heavy-duty coating method requires re-coating within 10–15 years, and that significantly increases the maintenance costs in semi-permanent steel structures; besides, it may include certain toxic elements in the applied coating [4]. Furthermore, the hot dip galvanizing method represents weak points, such as the size limitations of structural members, the thermal deformation of base materials and difficulties in welding after applying the coating in high-strength joint parts [5]. The hot dip galvanizing method generated detached coating layers with time and showed deterioration in the external appearance in outdoor applications due to partially contaminated black areas and chloride carbonic zinc [6].

In recent years, a thermal metal spraying method that produces an anti-corrosion layer on the surface of steel materials by melting Zn or Al, which has a relation to the sacrificial anode, using gas or electricity instead of the conventional corrosion resistance method, is attracting many researchers presently [7,8,9]. Thus, it is of great importance to select an appropriate thermal metal spraying method depending on the environmental conditions, as the anti-corrosion performance of the arc thermal metal spraying method (ATMSM) is highly influenced by the types of metals used [10,11]. In addition, the thermal spraying layer has a porous structure, and the characteristics of the anti-corrosion criteria vary with the process of applying the sealing coating that fills the pores [12]. Meanwhile, a quantitative study of this method with electrochemical experiments is needed, as conventional experiments, such as CASS (Copper ions accelerated salt spray) testing and salt spraying testing, have a qualitative limitation in the evaluation of the durability lifetime in ATMSM [13,14,15,16,17].

Furthermore, sacrificial anode metals, such as tin (Sn), magnesium (Mg) and indium (In), which have a better quality of corrosion resistance than zinc (Zn) or aluminum (Al), have been under extensive research study [18]. However, it seems that there are very few works that quantitatively evaluate the electrochemical performance of ATMSM using these metals [19,20,21].

The objective of the study is to quantitatively evaluate by the electrochemical technique the influence of the metal wire type and the presence of the epoxy sealing coating treatment of the sprayed layer on the anti-corrosion performance of ATMSM. 

## 2. Experiments

**Figure 1:**
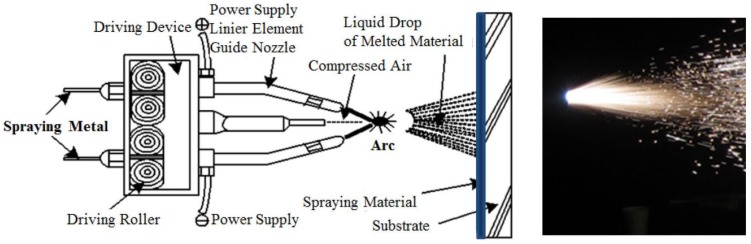
Arc thermal metal spraying method.

Table 1 shows the specimens that were used for the electrochemical experiments, which were conducted with the type of sprayed metal and the presence or absence of epoxy sealing coating. Pure zinc with a diameter of 1.6 mm, pure aluminum, Zn–Sn (65%:35% by mass (alloy)) and Al–Mg (95%:5% by mass (alloy)) were used as thermal spraying metals. Non-painted steel plates and hot dip galvanized steel plates produced at the factory were used for comparison. After blasting with grit, a thermal spray coating layer of a thickness up to 100 µm on the surface of steel plates is produced by using an arc thermal spraying gun, as shown in Figure 1 [7,8,9]. The Zn–Al thermal metal spraying method is a method that produces a corrosion resistance layer on the surface of steel materials using compressed air by melting metals, such as Zn and Al, with electric arcs. Figure 1 demonstrates an arc sprayer used in a Zn–Al thermal metal spraying method. The metal spraying discharges the metal melted at an arc point through a circular slit, and that can be introduced into an air stream in which it can be diffused and cooled. Then, the diffused metal generates a layer on the surface of steel by avoiding collisions and forms a porous metal spraying layer according to the accumulation and solidification of such layers. Test specimens (Zn 73%:Al 27%) sealed with epoxy coating after the formation of the thermal spray coating layer were also prepared.

**Table 1:** Specimens for the electrochemical experiments.

No.	Specimen Name	Specimens for Electrochemical Test	Epoxy Sealing Coating	Types of Spraying Metal	Anti-Corrosion Method
1	NP	Non-painted specimen	No	-	-
2	HDG	Hot dip galvanizing (zinc) 400 g/m^2^	No	-	Plating
3	Z100-NS	Zn (mass 100%)	No	Zn–Zn	Arc thermal metal spray
4	Z100-S	Zn (mass 100%)	Yes	Zn–Zn	
5	A100-NS	Al (mass 100%)	No	Al–Al	
6	A100-S	Al (mass 100%)	Yes	Al–Al	
7	Z73-A27-NS	Zn–Al (mass 27%)	No	Zn–Al	
8	Z73-A27-S	Zn–Al (mass 27%)	Yes	Zn–Al	
9	Z65-S35-NS	Zn–Sn (mass 35%)	No	Zn·Sn–Zn·Sn	
10	Z65-S35-S	Zn–Sn (mass 35%)	Yes	Zn·Sn–Zn·Sn	
11	A95-M5-NS	Al–Mg (mass 5%)	No	Al·Mg–Al·Mg	
12	A95-M5-S	Al–Mg (mass 5%)	Yes	Al·Mg–Al·Mg	-
Common items		Steel plate: SS41, 15 mm × 15 mm × 1.6 mm thickness; experimental area: 0.78 cm^2^ Steel surface treatment: grit blast			

A 15 mm × 15 mm specimen was connected to electric wires in which all spaces, except for 0.78 cm^2^, were sealed to prevent electric contacts. Corrosion potential and polarization resistance were measured through the exposed section of 0.78 cm^2^.

**Figure 2:**
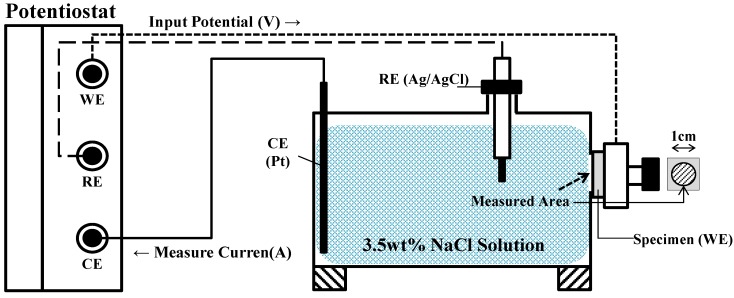
Schematic diagram for test setup. WE: working electrode; CE: counter electrode.

An electrochemical system with three electrodes, where the specimen was configured as a working electrode (WE), graphite was configured as a counter electrode (CE), and a silver-silver electrode (Ag/AgCl) was configured as a reference electrode, was used in this study. Figure 2 shows the setup for the electrochemical experiment. In addition, a VersaSTAT (Princeton Applied Research, Oak Ridge, TN, USA) was used to analyze the results of these experiments.

Changes in potential and currents were measured with a 1.0-mV/s projection speed in the range of −0.4–+0.8 mV based on the corrosion potential. Then, the polarization resistance was observed by calculating the obtained potential. The solution used in this experiment was a 3.5 wt% NaCl solution at 25 °C.

Table 2 shows sectional images produced using an SEM (scanning electron microscopy). In the case of the hot dip zinc galvanizing method, it shows a very dense, uniform and high adhesive layer that enables the specimen to have very good corrosion resistance in steel structures based on the sacrificial anode of Zn.

In the case of the specimens that were coated using pure Zn (No. 3) and Al (No. 4) by ATMSM, it appears that there is a rough surface accompanying many open holes. There are also some pores and micro-cracks in the image morphology, but these pores and cracks were not connected with each other nor traversing the coating from the coating surface to the steel substrate.

For the specimen with Zn–Al thermal metal spray (No. 5), the melted Zn (dark color) and Al (bright color) accumulated and formed a dense pseudo-alloy coating on the surface of the steel plates. A similar observation can be seen in specimen No. 6, too. As for Zn–Sn (65%:35%) in the mass specimen, there is rough surface accompanying many open holes. There are also many pores and micro-cracks in the image morphology. Specimen No. 8 shows a clear rough surface accompanying open holes, and also, there are some pores with a relatively large size.

From these SEM images, one can see that, based on the principal of a sacrificial anode, specimens coated with Zn, Al, Zn–Al, Zn–Sn and Al–Mg by ATMSM have a relatively high potential to resist corrosion.

**Table 2:** SEM (scanning electron microscopy) images for each corrosion resistance method.

No.	Specimen Name	SEM Image
1	NP	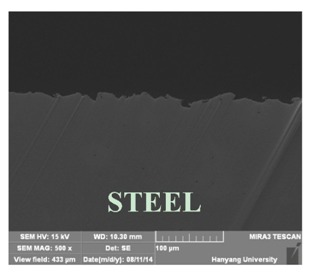
2	HDG	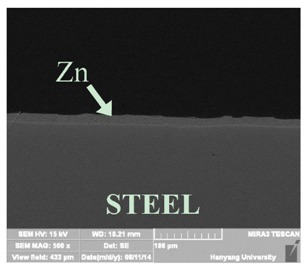
3	Z100-NS	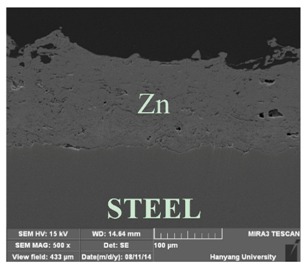
4	A100-NS	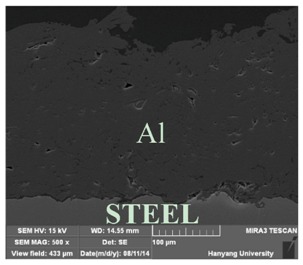
5	Z73-A27-NS	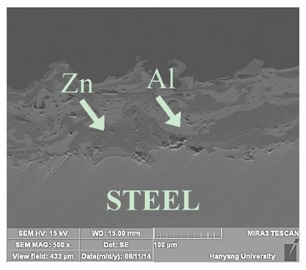
6	Z73-A27-S	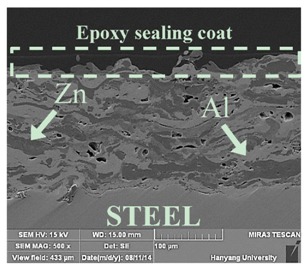
7	Z65-S35-NS	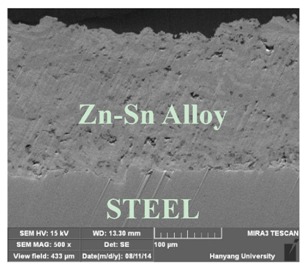
8	A95-M5-NS	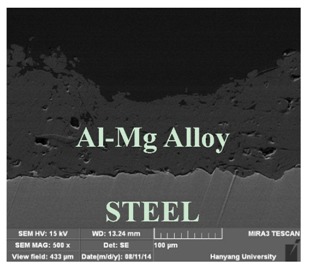

## 3. Discussion

### 3.1. Experimental Results

Table 3 shows the results of the electrochemical tests. The corrosion potential of the non-painted specimen was −0.61 V and the other specimens showed more negative corrosion potential. Thus, the sacrificial anodic reaction of the sprayed metal made the steel plate effectively anti-corroded in HDG and metal spray specimens. It is also seen in this table that the comparison of the corrosion potential values of all specimens in relation to HDG suggests that epoxy sealing coating contributes dramatically to the corrosion resistance performance of these specimens coated with ATMSM.

**Table 3:** Electrochemical test results.

No.	Specimen Name	Open Circuit Potential (V)	Corrosion Potential (V)	Relative Corrosion Rate (HDG = 1)
1	NP	−0.55	−0.61	0.30
2	HDG	−0.92	−0.91	1.00
3	Z100-NS	−1.03	−1.25	2.27
4	Z100-S	−0.98	−0.96	0.98
5	A100-NS	−0.99	−1.06	0.34
6	A100-S	−0.10	−1.02	0.01
7	Z73-A27-NS	−1.05	−1.23	0.19
8	Z73-A27-S	−0.98	−0.97	0.02
9	Z65-S35-NS	−1.03	−1.30	0.66
10	Z65-S35-S	−0.97	−0.98	0.16
11	A95-M5-NS	−0.86	−1.07	0.18
12	A95-M5-S	−0.80	−0.91	0.001

Table 4 shows the corrosion conditions on the surface of the specimens before and after the electrochemical experiment. The surface of the non-painted (NP) specimen was significantly corroded. In particular, the corrosion of the metal spraying layer on the surface of the specimens using Zn and Sn, the ionization tendencies of which are generally higher than iron, were remarkably observed. The metal spraying layer in specimen Z73-A27-NS, in which the holes were not filled with the epoxy sealing coating, was partially corroded. On the other hand, for the specimens that were coated with epoxy sealing coating, the corrosion of the layer is hardly observed. From these observations, one can see that epoxy sealing coating has an obvious effect on the anti-corrosion performance of thermally-sprayed specimens.

**Table 4:** Surface conditions of the specimens before and after carrying out the electrochemical experiment.

No.	Specimen Name	Before Exp.	After Exp.	No.	Specimen Name	Before Exp.	After Exp.
1	NP	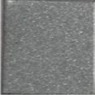	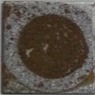	7	Z73-A27-NS	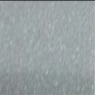	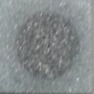
2	HDG	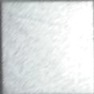	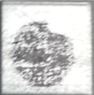	8	Z73-A27-S	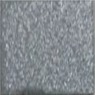	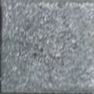
3	Z100-NS	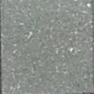	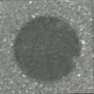	9	Z65-S35-NS	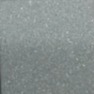	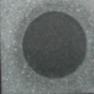
4	Z100-S	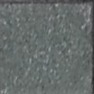	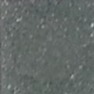	10	Z65-S35-S	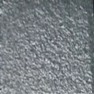	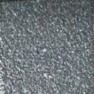
5	A100-NS	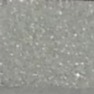	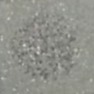	11	A95-M5-NS	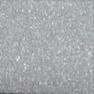	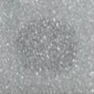
6	A100-S	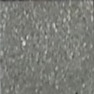	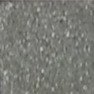	12	A95-M5-S	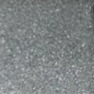	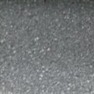

### 3.2. Open Circuit Potential and Corrosion Potential

**Figure 3:**
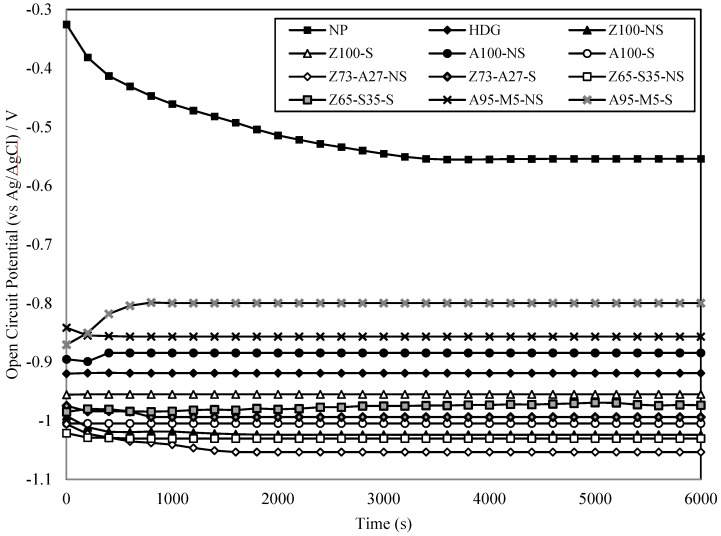
Relationship between open circuit potential and time.

Figure 3 illustrates the value of open circuit potentials produced in a very short time in the application of ATMSM. The open circuit potential of the NP specimen was found to be stable at about −0.55 V (*vs*. Ag/AgCl). However, HDG and metal-sprayed specimens showed an open circuit potential between −0.9 V (*vs*. Ag/AgCl) and −1.0 V (*vs*. Ag/AgCl), and that value varied depending on the type of sprayed metal. Therefore, ATMSM was found to exhibit a sufficient sacrificial anode effect for the corrosion resistance of steel.

**Figure 4:**
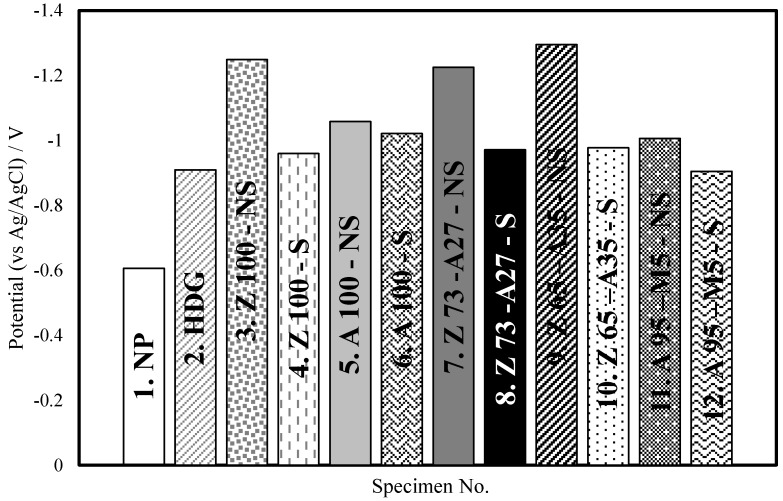
Corrosion potential in each specimen.

Figure 4 shows the stable corrosion potential (*E*_corr_) of each tested specimen. The corrosion potential (*E*_corr_) of specimen NP was –0.606 V (*vs*. Ag/AgCl). The corrosion potential of metal-sprayed and HDG specimens ranges from –0.9 to –1.25 V (*vs*. Ag/AgCl), indicating a difference of about –0.6 V (*vs*. Ag/AgCl). Therefore, ATMSM in this experiment is thought to produce an excellent galvanic effect, because the corrosion potential difference of –0.1 V (*vs*. Ag/AgCl) is generally sufficient. Because Zn is more vulnerable to corrosion than other metals, specimens coated with Zn showed a negative potential, which is relatively higher than those of other metals.

### 3.3. Polarization Curve

**Figure 5:**
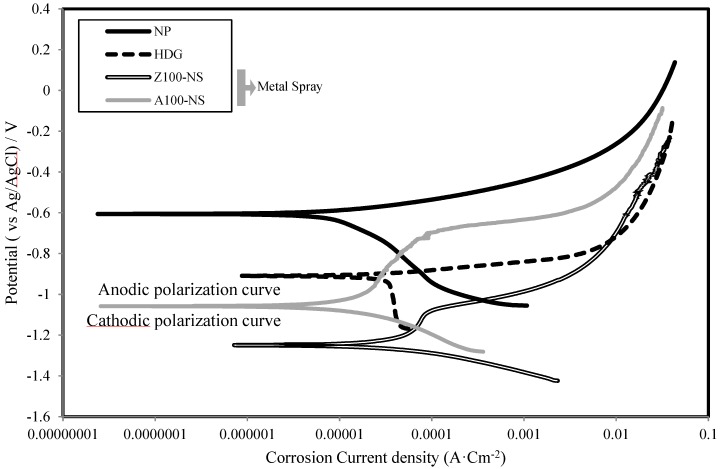
Polarization curves of NP, HDG, Z100-NS and Al100-NS specimens.

Figure 5 shows the polarization curves of specimens in the case of NP, HDG, Z100-NS and Al100-NS. It is clear that there are high potential differences between the coated specimens and non-painted specimen. In the anodic polarization area, the anodic reaction of the HDG specimen using Zn is as follows: Zn + 2Cl^−^ → ZnCl_2_ + 2e^−^. The melting reaction proceeds rapidly, because the polarization curve is close to being horizontal. This is similar to the metal spraying of Zn. The minimum −1.25 V indicates that dissolution of Zn takes place actively and that the galvanic effect is excellent. Stagnation of the current increase near the corrosion current density of 0.0001 A·cm^−2^ was partially observed. Because only the top surface of the plates was treated with epoxy sealing coating, corrosion had occurred in some part of the steel surface. On the other hand, specimen Al100-NS showed a corrosion potential of −1.06 V. The corrosion current density was in a steady state at about 0.0001 A·cm^−2^, and it increased rapidly, primarily due to the formation of oxide film (Al + 3OH^−^ → Al(OH)_3_ + 3e^−^) and of corrosion products (Al + 3Cl^−^ → AlCl_3_ + 3e^−^). In the cathodic polarization area, the current in the HDG specimen was in a steady state at about 0.0001 A·cm^−2^, which suggests that the surface of the electrode was coated with Zn(OH)_2_ film at the beginning of polarization. On the other hand, the corrosion current density of specimens Al100-NS and Z100-NS increased steadily, and dissolutions of the metal proceeded smoothly.

**Figure 6:**
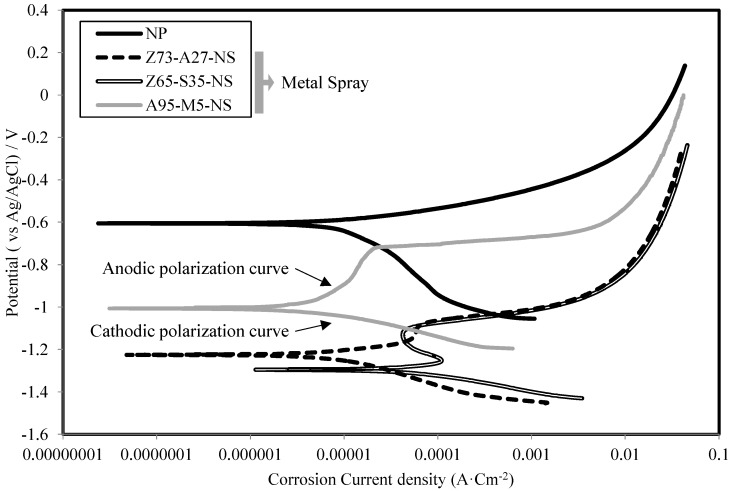
Polarization curves of NP, Zn–Al, Zn–Sn and Al–Mg specimens.

Figure 6 shows the polarization curves of NP, HDG, Z100-NS and Al100-NS specimens. The corrosion potential was about −1.23 V in the specimen with the pseudo-alloy of Zn–Al. In the anodic and cathodic polarization areas, the melting of Zn occurred easily with a steady increase of the corrosion current density. The corrosion potential of the Zn–Sn alloy specimen had a minimum of −1.30 V, and the corrosion current density was stabilized at 0.0001 A·cm^−2^ in the anodic area. Following the melting of Sn with high solubility, the rapid dissolution of Zn occurred. Hence, corrosion occurred on the steel surface. On the other hand, the Al-Mg alloy specimen showed a −1.07 V corrosion potential. The corrosion current potential was stabilized at 0.000001 A·cm^−2^ in the anodic polarization area. This is because aluminum hydroxide coating was formed after the dissolution of Mg.

**Figure 7:**
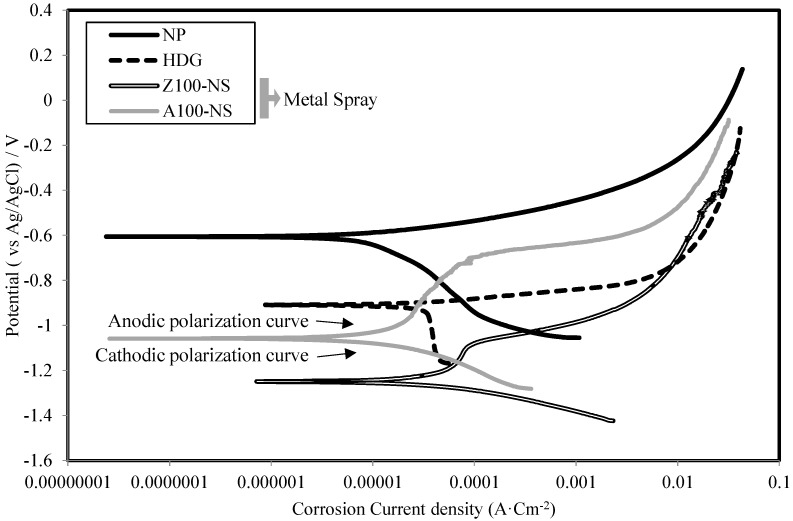
Polarization curves of Z73-A27 with and without epoxy sealing coating.

Figure 7 shows the polarization curve of Zn–Al and Al–Mg specimens without and with epoxy sealing coating. The corrosion potential of the Z73-A27-NS specimen, which was not coated with epoxy sealing coating, is −1.23 V. This is higher than that of the corrosion potential of the Z73-A27-S specimen of −0.970 V, in which epoxy sealing coating was applied.

The reason for this may be due to the fact that the epoxy sealing coating obstructs the dissolution of Zn–Al. The corrosion current density stabilization at about 1.0 × 10^−5^ A·cm^−2^ verifies that there was an obstruction from the epoxy sealing coating, and this is clear from the cathodic polarization area of the specimen with epoxy sealing coating.

A similar observation can be seen in the case of Al–Mg, and the effect of epoxy sealing coating in this case is significant and makes it obvious that this combination of Al–Mg gives the best results in comparison with the other metals used so far.

### 3.4. Corrosion Current Density and Polarization Resistance

The potentiodynamic polarization curve, known as a Tafel plot, depicts the relationship between potential and corrosion current density. This plot exhibits a linear region, the slope of which is known as the Tafel constants (anodic and cathodic Tafel constants). The intersection of the projection of the linear region of the plot with the open circuit potential (*E*_corr_) gives the cathodic or anodic corrosion current (*i*_corr_). Once *i*_corr_ is determined, the following equation, derived from Faraday’s law, can be used to calculate the corrosion rate [22]:

(1) $$\text{Corrosion rate }\left(\text{μm}/y\right)=\;\frac{3.27\;\;\times \;I_{\text{corr}}\;\times \;E.W.}{d}$$

The corrosion rate in Equation (1) is expressed in micrometers per year, μm/y. *I*_corr_ is the corrosion current density in µA·cm^−2^, obtained by dividing *i*_corr_ with the exposed surface area of the measured specimen. *E.W.* is the equivalent weight of steel in g, and *d* is the density of steel in g/cm^3^.

The polarization resistance *R*_p_ (Δ*E*/Δ*I*), which is the slope of the potential-current curve at *E*_corr_, is related to *I*_corr_ through the following Stern–Geary relationship [23]:

(2) $$I_{\text{corr}}\;\left(\text{μA}/{\text{cm}}^{2}\right)=\;\frac{\left[\beta_{a}\;\times \;\beta_{c}\right]}{2.3\;(\beta_{a}\;+\;\beta_{c})}\;R_{p}$$

ß_a_ and ß_c_ are the anodic and the cathodic Tafel constants, respectively, expressed in mV/decade of the current. *R*_p_ is expressed in KΩ·cm^2^. It is seen here that for the determination of *I*_corr_ in this technique, ß_a_ and ß_c_ are determined from the Tafel plot.

Table 5 shows the corrosion current density (*I*_corr_), corrosion rate (CR) and polarization resistance (*R*_p_) values of each specimen’s type. The polarization resistance of specimen Z73-Al27-S, with the application of the epoxy sealing coating, was 11.45 KΩ·cm^2^, which is about four-times that of specimen Z73-Al27-NS (2.63 KΩ·cm^2^) without the application of the epoxy sealing coating. Thus, the effect of corrosion resistance by the epoxy sealing coating is clear. One can also see that the polarization resistance of specimen A95-M5-S, with the application of the epoxy sealing coating, was 5,255.64 KΩ·cm^2^, which is almost 650-times that of the same specimen without epoxy sealing coating. Similar outcomes can be seen from the corrosion rate and corrosion current density results. These results confirm that specimen Al:Mg (95%:5%) produces the best anti-corrosion performance in this present study.

**Table 5:** Values of corrosion current density, corrosion rate and polarization resistance of each type of specimen.

No.	Specimen Name	Corrosion Current Density (µA·cm^−2^)	Corrosion Rate (µm/y)	Polarization Resistance (KΩ·cm^2^)
1	NP	11.30	132.48	2.30
2	HDG	29.10	439.18	0.50
3	Z100-NS	65.90	996.33	0.35
4	Z100-S	28.4	429.02	0.74
5	A100-NS	13.60	149.87	2.46
6	A100-S	0.2	2.26	521.43
7	Z73-A27-NS	7.91	111.49	2.63
8	Z73-A27-S	0.63	8.85	11.45
9	Z65-S35-NS	17.80	289.82	1.07
10	Z65-S35-S	4.28	69.81	3.07
11	A95-M5-NS	7.02	79.54	28.09
12	A95-M5-S	0.02	0.29	5,255.64

## 4. Conclusions

Based on the results of this study, the following conclusions may be drawn:

1. The sacrificial anode principle in ATMSM was verified using an electrochemical technique. This is evident from the low values of corrosion current density (*I*_corr_) and high values of polarization resistance (*R*_p_) of specimens with sprayed metal, such as Zn, Al, Zn–Al, Zn–Sn and Al–Mg, compared with Fe alone.
2. It is obvious that the corrosion resistance layer generated by applying ATMSM provided a sufficient corrosion resistance with the driving force based on the potential difference of more than approximately 0.60 V between the layer and the base material.
3. The corrosion potential difference of the specimens in regards to the presence or absence of epoxy sealing coating in the specimens of Zn–Al metal thermal spraying is about 0.25 V, while in the case of Al–Mg, it is 0.16 V. Thus, the effect of the corrosion resistance of steel by epoxy sealing coating suppresses the dissolution of metals, decreases the corrosion current density and increases the polarization resistance.
4. The results of the metal thermal spraying without applying the epoxy sealing coating show that the corrosion current density of the specimen using Al–Mg (95:5) is 7.02 µA·cm^−2^; the corrosion rate is 79.54 µm/y; and polarization resistance is 28.09 K Ω·cm^2^. These values in the case of applying the epoxy sealing coating are 0.02 µA·cm^−2^, 0.29 µm/y and 5255.64 KΩ·cm^2^, respectively. This is clear evidence that this type of metal thermal spraying has the best performance and exceeds even the Zn–Al type.
